# Accurate HLA type inference using a weighted similarity graph

**DOI:** 10.1186/1471-2105-11-S11-S10

**Published:** 2010-12-14

**Authors:** Minzhu Xie, Jing Li, Tao Jiang

**Affiliations:** 1 Department of Computer Science and Engineering, University of California, Riverside, CA 92521, USA; 2 College of Physics and Information Science, Hunan Normal University, Changsha 410081, P. R. China; 3 Department of Electrical Engineering and Computer Science, Case Western Reserve University, Cleveland, OH 44106, USA

## Abstract

**Background:**

The human leukocyte antigen system (HLA) contains many highly variable genes. HLA genes play an important role in the human immune system, and HLA gene matching is crucial for the success of human organ transplantations. Numerous studies have demonstrated that variation in HLA genes is associated with many autoimmune, inflammatory and infectious diseases. However, typing HLA genes by serology or PCR is time consuming and expensive, which limits large-scale studies involving HLA genes. Since it is much easier and cheaper to obtain single nucleotide polymorphism (SNP) genotype data, accurate computational algorithms to infer HLA gene types from SNP genotype data are in need. To infer HLA types from SNP genotypes, the first step is to infer SNP haplotypes from genotypes. However, for the same SNP genotype data set, the haplotype configurations inferred by different methods are usually inconsistent, and it is often difficult to decide which one is true.

**Results:**

In this paper, we design an accurate HLA gene type inference algorithm by utilizing SNP genotype data from pedigrees, known HLA gene types of some individuals and the relationship between inferred SNP haplotypes and HLA gene types. Given a set of haplotypes inferred from the genotypes of a population consisting of many pedigrees, the algorithm first constructs a weighted similarity graph based on a new haplotype similarity measure and derives constraint edges from known HLA gene types. Based on the principle that different HLA gene alleles should have different background haplotypes, the algorithm searches for an optimal labeling of all the haplotypes with unknown HLA gene types such that the total weight among the same HLA gene types is maximized. To deal with ambiguous haplotype solutions, we use a genetic algorithm to select haplotype configurations that tend to maximize the same optimization criterion. Our experiments on a previously typed subset of the HapMap data show that the algorithm is highly accurate, achieving an accuracy of 96% for gene HLA-A, 95% for HLA-B, 97% for HLA-C, 84% for HLA-DRB1, 98% for HLA-DQA1 and 97% for HLA-DQB1 in a leave-one-out test.

**Conclusions:**

Our algorithm can infer HLA gene types from neighboring SNP genotype data accurately. Compared with a recent approach on the same input data, our algorithm achieved a higher accuracy. The code of our algorithm is available to the public for free upon request to the corresponding authors.

## Introduction

In human chromosomal region 6p21, there is a Human Leukocyte Antigen (HLA) super-locus of about 4Mb length with extreme high levels of gene density and variation. The HLA locus contains about 0.5% (> 150) of all known protein coding genes [1] and nearly each HLA gene has more than a dozen different alleles [2]. The HLA genes play important roles in the immune system and encode a group of related proteins known as the HLA complex. The highly polymorphic HLA genes produce hyper variable HLA complex, by which the human immune system differentiates self cells and non-self cells. Mismatches between an organ donor’s HLA genes and a recipient’s HLA genes usually result in rejection reactions and cause a transplantation to fail. The nature of the genetic diversity of the HLA region is complex, and this region has become a research hot-spot in human genomics [3]. Recently, numerous researchers have illustrated that different alleles of HLA genes are associated with many autoimmune, inflammatory and infectious diseases [4,5]. However, direct experimental typing methods of HLA gene alleles such as serology and PCR are laborious, time consuming and expensive, which limit large-scale studies concerning HLA genes [6]. So, effective computational techniques are in demand to help determining HLA gene types.

**Figure 1:**
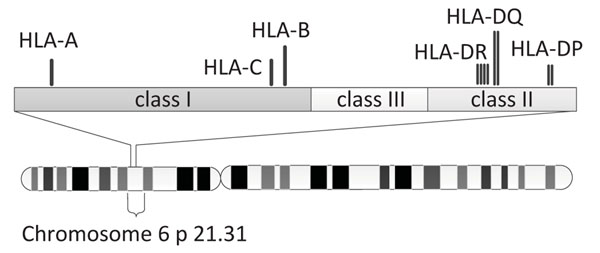
HLA region of chromosome 6

**Figure 2:**
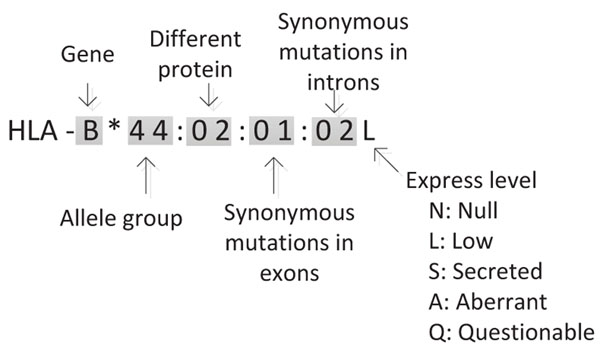
HLA nomenclature

The HLA region is divided into two classical regions called class I and class II, and an intervening region denoted as class III, as illustrated in Figure 1. The classical genes HLA-A, HLA-B and HLA-C are in Class I; and the classical genes HLA-DP, HLA-DQ and HLA-DR are in Class II [3]. There is an elaborate nomenclature for hyper variable HLA gene alleles, as shown in Figure 2. Beginning with HLA- and the gene name, each HLA allele name contains up to four sets of digits separated by colons. The first set of digits describes the allele group, which can be determined by serological typing. The next set of digits describes the subtypes and represents different amino acid sequences of the encoded protein. The last two sets of digits denote any synonymous mutations in exons and introns respectively. An additional optional suffix such as ‘L’, ‘S’, ‘C’, ‘A’ or ‘Q’ is used to specify its expression level or other non-genomic data. Although four sets of digits are needed to completely describe an allele, most practical applications usually only require the first set or the first two sets of digits.

With the advance of high throughput SNP genotyping technologies, it is relatively easy to obtain genome-wide SNP genotype data with low costs, and SNP data of many individuals has already been available. Recently, some researchers have studied the problem of HLA gene type inference based on SNP genotype data. The first type of approaches are rooted in the concept of tag SNPs. Based on the linkage disequilibrium between multiallelic HLA genes and their neighboring biallelic SNPs, de Bakker *et al.*[4] selected up to three tag SNPs as predictors of HLA alleles. Although tag SNP based methods in general can infer some common HLA alleles [7-9], they usually select different sets of tag SNPs for different alleles of the same HLA gene. Moreover, HLA genes are highly polymorphic and the majority alleles are rare. It is known that they generally cannot be distinguished by using combinations of up to three tag SNPs [6]. By extending the above tag SNPs based methods, Leslie *et al.*[6] selected dozens of SNPs around the HLA genes and proposed a statistical method to infer HLA alleles at class I and class II loci. The method is based on the assumption that a chromosome carrying an HLA allele is an imperfect mosaic of those chromosomes with the same HLA allele. Given a training data of SNP haplotypes and the corresponding phased HLA gene types, a hidden Markov model is used to calculate the posterior probability of a chromosome taking an HLA allele with a particular SNP haplotype. The model requires a fine genetic map of the region [6], and uses a training set of SNP haplotypes with known, phased HLA gene types [2]. Based on the identity by descent (IBD) information between pairs of individuals, Setty *et al.*[2] proposed an iterative approach for HLA type imputation. At first, a program (GERMLINE [10]) is called to obtain the IBD segments between each pair individuals and an IBD-Graph is built for the individuals with known or unknown HLA types. Then the unknown HLA types of some individuals are imputed from the individuals with known HLA types that are involved in the same triplets of the IBD-Graph. At last the IBD-Graph is updated and a new iteration begins until no more HLA types can be imputed. Though the IBD-Graph based method does not require SNP haplotypes as a part of the input, the program GERMLINE [10] needs SNP haplotype information to determine the IBD status between each pair of individuals.

The accuracy of all above methods critically depends on the accuracy of the haplotypes of each individual, which are usually inferred from genotypes based on some computational models. Though the haplotype inference problem from unrelated individuals has been extensively studied recently [11], the accuracy of inferred haplotypes, especially for large chromosome regions (> 100 kb), is not yet satisfactory [12]. Additional information from family data could greatly improve the accuracy of haplotype inference for long chromosomal regions [13,14]. However, even with pedigree information, there are still potentially many haplotype solutions for a pedigree that satisfy the Mendelian law and have the smallest number of recombination events [13,14].

In this paper, we jointly model HLA gene type inference and SNP haplotype inference/selection within one unified framework, by utilizing the relationship among individuals (*i.e.*, pedigree information), known HLA gene types of some individuals and the relationship between SNP haplotypes and HLA gene types. We first propose a new haplotype similarity measure and construct a weighted haplotype similarity graph. Known HLA gene types are used to derive additional constraints on edges connecting two haplotypes from the same individual. Based on the principle that different HLA gene types should have different background SNP haplotypes, the algorithm searches for an optimal labeling of all the haplotypes with HLA gene types such that the total weight among the same HLA gene types is maximized. To obtain haplotypes from genotypes, we first utilize the program recently developed in [14] to construct a solution space with the minimum number of recombinants from each pedigree. To deal with ambiguous haplotype solutions for each pedigree, an enumerating procedure is used to select a haplotype configuration that tends to maximize the total similarity among the same HLA gene types. When there are too many solutions for the enumerating procedure to work efficiently, a genetic algorithm is adopted instead. Compared with the existing methods, our algorithm achieves a higher accuracy on a previously studied HapMap dataset.

## Results and discussion

The performance of our algorithm (denoted as WSG-HI) is evaluated using the dataset from [2], which can be downloaded from http://www.inflammgen.org/inflammgen/files/data/. The data consists of 180 Utah residents of European ancestry (27 extended families with an average family size of 6.6) from the CEPH collection (CEU) of the HapMap project, which has been described in [4]. 8562 nonredundant variants in the 7.5-Mb extended HLA region were genotyped [4], of which 6300 SNPs passed QC [6]. The HLA typing for three class I genes (HLA-A, HLA-B, and HLA-C) and three class II genes (HLA-DRB1, HLA-DQA1, and HLADQB1) was carried out with PCR-SSOP protocols. Both the SNP data and HLA gene data used in our experiments are unphased and were obtained from [4].

Two important measures *coverage* and *accuracy* are used to analyze and compare the performance of different algorithms, which have also been used in [2]. They are defined as follows(1)(2)

where *N_analyzed_* denotes the number of chromosomes analyzed by each algorithm, *N_called_* denotes the number of chromosomes whose HLA genes have been inferred, and *N_correct_* denotes the number of chromosomes whose HLA genes have been correctly inferred.

### Sketch of the algorithm and experimental design

**Figure 3:**
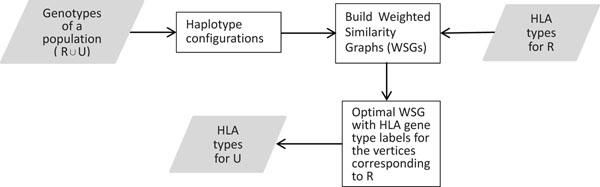
A sketch of WSG-HI

A brief sketch of WSG-HI is illustrated in Figure 3. The details of WSG-HI are described in Section **Methods**. The input data are the unphased SNP genotype data of a population **P** and the known unphased HLA gene types of the individuals in the subset R. The algorithm outputs the inferred HLA gene types for the individuals in the subset **U=P–R**.

We will use two test strategies: leave-one-out and leave-one-pedigree-out. In the leave-one-out test, for each individual in the CEU data, its HLA gene types are removed and WSG-HI is used to infer the HLA gene types of this member. In the leave-one-pedigree-out test, for each pedigree in the population, the HLA gene types of all members of the pedigree are removed and the algorithm is used to infer the HLA gene types of every member of the pedigree.

### Results of the leave-one-out test

**Figure 4:**
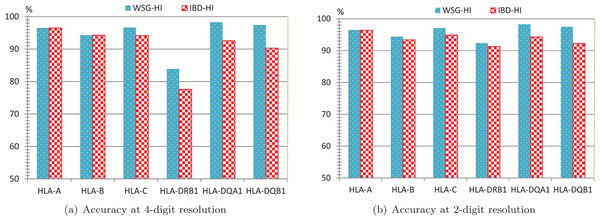
**Comparison with IBD-HI** Comparison of the algorithm of Setty *et al.* (labeled as IBD-HI) and our algorithm (labeled as WSG-HI) a both 4-digit (a) and 2-digit (b) resolution levels. The accuracy of IBD-HI is obtained directly from [2], an all results are based on the leave-one-out test using genotype data from the 200kb regions centered around each HLA gene.

We download the same data set used in Setty *et al.*[2], and compare the performance of our algorithm (WSG-HI) and theirs (denoted as IBD-HI). To evaluate the accuracy, both algorithms use the leave-one-out test method as described in [2]. For each HLA gene, WSG-HI takes the genotype data from the region of 200kb centered around the HLA gene under consideration (*i.e.*, the region spanning 100kb upstream and downstream of the gene) as the input. The results at two different HLA gene allele resolution levels (4-digit and 2-digit) are illustrated in Figure 4. HLA gene types that are not resolved to the required resolution level or occur just once in the data are excluded from the analysis. Since the coverage of WSG-HI for all HLA genes is 100% in the experiments and Setty *et al.* did not provide the coverage of their algorithm in [2], we only compare the accuracy of both algorithms. As shown in Figure 4, both algorithms perform similarly for HLA-A and HLA-B. However, for the other four genes, WSG-HI is more accurate than IBD-HI at both 4-digit and 2-digit resolutions.

**Figure 5:**
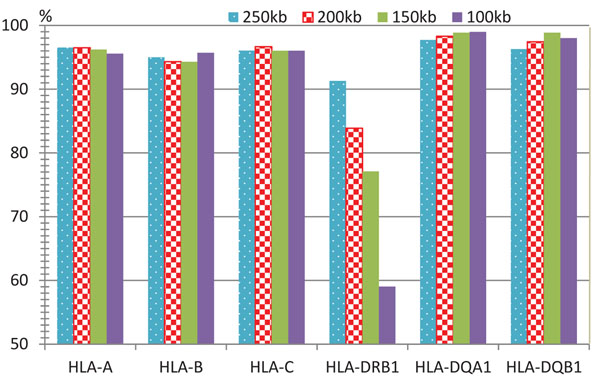
**The accuracy of GSW-HI** When the size of the genotype region used by the algorithm changes, the accuracy of GSW-HI varies slightly with the exception of HLA-DRB1.

We also investigate how the size of the region around an HLA locus, where the SNP genotype information is used by WSG-HI, affects the HLA gene type inference accuracy. Figure 5 illustrates that when the size of SNP genotype region changes from 250kb to 100kb, the accuracy of WSG-HI varies slightly for almost all the genes, with the only exception of the HLA-DRB1 gene. For HLA-DRB1, WSG-HI achieves the highest accuracy of 91.3% when using genotype data from the 250kb region surrounding the gene, and its performance deteriorates dramatically when the size of the region gets smaller. This is mainly caused by the fact that the number of heterozygous SNPs near the center of the HLA-DRB1 gene region is very small (see Additional file 1). Therefore, when using the region of 100kb, there are not enough SNP haplotypes to distinguish all different HLA-DRB1 gene types.

### Results of the leave-one-pedigree-out test

Because the test data consists of many pedigrees, it is conceptually easier to infer the HLA gene types of a tested member in the above leave-one-out strategy. The known HLA gene types of other members in the same pedigree may provide much needed information to correctly infer HLA gene types of the tested member. Therefore, we further test the performance of our algorithm using the leave-one-pedigree-out strategy, by deleting the HLA gene type information of a whole pedigree. The experimental results are shown in Table 1. To our surprise, the results are almost as good as those from the leave-one-out strategy. This is probably because the haplotype similarity information at the population level has already provided sufficient information to correctly infer HLA gene types. One does not gain much from additional information provided by family members, especially in our experimental settings when such relationship is not explicitly explored.

**Table 1:** Experimental results of the leave-one-pedigree-out test using genotype data from the 200k region centered around each HLA gene.

Gene	4-digit		2-digit	

	*Coverage*(%)	*Accuracy*(%)	*Coverage*(%)	*Accuracy*(%)
HLA-A	100	95.6	100	95.3
HLA-B	100	93.2	100	93.0
HLA-C	100	95.6	100	95.3
HLA-DRB1	100	80.8	100	92.4
HLA-DQA1	100	93.5	100	94.9
HLA-DQB1	100	94.0	100	94.2

## Conclusions

HLA genes have important functions in the human immune system, and their variations are associated with many complex diseases. However, directly typing of HLA genes is time consuming and expensive. Accurate and efficient computational algorithms to infer HLA gene types from SNP genotype data are a good alternative. We have designed an accurate HLA gene type inference algorithm (WSG-HI). The algorithm takes SNP genotypes of all individuals and HLA gene types of a subset of individuals as input, and infers the HLA gene types of the remaining individuals based on their genotype data. Extensive experimental results on a previously typed dataset have illustrated that WSG-HI can infer the HLA gene types from their neighboring SNP genotypes accurately. Compared with a previous approach based on IBD, our algorithm achieves the same accuracy for HLA-A and HLA-B genes, but is much more accurate for HLA-C, HLA-DRB1, HLA-DQA1 and HLA-DQB genes.

## Methods

### Preliminaries

A *single nucleotide polymorphism* (SNP) is the change of a single nucleotide at a position of the genome sequence and is the major form of human genome variation. It is believed that most SNPs are bi-allelic. Therefore, an SNP can usually be represented as a 0 or 1, where ‘0’ denotes the major allele at the SNP locus and ‘1’ denotes the minor allele. When the allele of an SNP is unknown, ‘–’ is used. A sequence of SNP alleles on one of a pair of homologous chromosomes is called a *haplotype*, and can be denoted by a string over {0, 1, –}. Two conflated (unordered pair of) SNP alleles at each SNP locus of a pair of homologous chromosomes is called a *genotype.*

**Figure 6:**
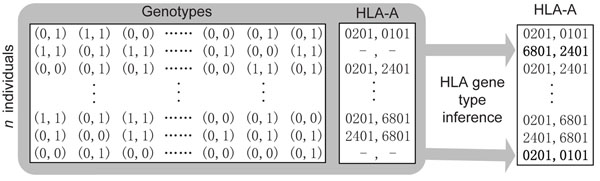
An example of the HLA gene type inference problem

Recently, extensive studies have revealed that bi-allelic SNPs in the HLA region are in strong linkage disequilibrium with multi-allelic HLA genes [4], which implies that similar haplotypes will harbor similar HLA gene alleles. However, determining haplotypes using biological techniques is as costly and time consuming as direct HLA gene typing. Because it is relatively inexpensive to genotype large-scale SNPs, inferring HLA gene alleles from their neighboring SNPs genotypes offers an attractive alternative to conventional HLA typing. For an HLA gene, given the genotype data around the HLA gene of a population and the HLA gene alleles of some individuals of the population, the *HLA gene type inference problem* aims to determine the HLA gene alleles (or types) for the individuals whose HLA gene alleles are unknown. Please see Figure 6 for an example. The genotype data of *m* SNPs of *n* individuals can be represented by an *n* × *m* matrix (called the genotype matrix) with each element representing an unordered SNPs pair, and the HLA gene types of the population can be denoted as an *n* × 2 matrix (called the HLA matrix) where an unknown gene type is considered as an empty element and denoted by ‘–’. In Figure 6 (right), the HLA-A gene types of the second and the last individuals are inferred using a computational approach. Although there are many different methods to infer haplotypes from genotype data, the accuracy of haplotype inference from a population made up of unrelated individuals is unsatisfactory. On the other hand, with additional family information, long range haplotypes can be inferred more accurately [11]. This is why Leslie *et al.*[6] and Setty *et al.*[2] chose pedigrees as their test data. However, even if a pedigree is provided, it may be difficult to uniquely determine its haplotype configuration [14]. In Li *et al.*[14], the authors designed a program *DSS* that could establish the solution space of the haplotype configurations of a pedigree under the Mendelian and zero-recombinant constraints in almost linear time. DSS uses a disjoint-set structure *D* to represent a general solution. The number of total specific solutions (*N_s_*) has a simple relationship with the number of free variables *f*: *N_s_* = 2*^f^*. To deal with recombination, DSS was extended as follows. At first, the whole region is partitioned into some maximal zero-recombinant segments. Then the solution spaces for these segments are combined into a whole solution space for the region with of the goal of minimizing the number of recombinations between neighboring segments. We adopt the DSS algorithm in this paper due to its efficiency and accuracy.

### Algorithm

Given the SNP genotypes of some pedigree data and the known HLA gene type information of some individuals, our algorithm jointly models the HLA gene type inference problem and the optimal haplotype selection problem within one framework. In brief, we first define a new haplotype similarity measure. Given a set of haplotypes, we construct a complete weighted graph with each node representing one haplotype. Each edge, connecting a pair of haplotypes, is given a weight using the value of the similarity between the two haplotypes. In addition, for each individual with known HLA types, a constraint edge is added between its two haplotypes and is labeled using its HLA gene types. Given such a graph, our goal is to search for an optimal assignment of each node using HLA gene types that satisfies all the edge constraints and maximizes the similarity measures between the haplotypes with the same HLA gene types. Because there are potentially multiple haplotype solutions for each pedigree, our algorithm will pick solutions that tend to maximize the overall similarities of haplotypes with the same HLA gene types. More specifically, the algorithm WSG-HI mainly consists of two steps. The first step is to search for an optimal relationship between HLA gene types and their background haplotypes, and at the same time, select a unique haplotype configuration for each pedigree from its multiple solutions. The second step is to compute an optimal assignment of HLA gene types for the individuals whose HLA gene types are unknown. For simplicity, the relationship between HLA gene types and their background haplotypes is called the *Hap-HLA relation*.

In the first step, the extended DSS [14] is applied to establish the solution space of the haplotype configurations of each pedigree in a population *P*. In many cases, the whole solution space of *P* may be too large to directly enumerate. For example, though most families of the CEU population have no more than 1024 different haplotype solutions, some pedigree have more than 2^60^ solutions (see Additional file 2). Therefore, an incremental method is adopted to construct the similarity graph. We first pick those pedigrees whose solution spaces contain only a unique solution to form a haplotype subpopulation *P*^′^, and a partial Hap-HLA relation is established based on the graph built from *P*^′^. Then the remaining pedigrees are sorted in the ascending order according to the size of their solution space and are precessed sequentially in the following manner. When the solution space of a pedigree is small, every haplotype solution for the pedigree is enumerated and combined with the current similarity graph to generate a new partial Hap-HLA relation. The optimization criterion is evaluated and the solution with the highest value is selected as the solution of this pedigree. When the number of haplotype solutions of a pedigree is too large to enumerate, a genetic algorithm is adopted. The process continues until all pedigrees have been added. Finally, in step two, unknown HLA allele types can be inferred based on the complete similarity graph by using the Hap-HLA relation established in the first step. The details of the algorithm are described in the remainder of this subsection.

#### Similarity between two haplotypes

Let *h_i_* = (*s_i_*_1_, *s_i_*_2_, …, *s_in_*) and *h_j_* = (*s_j_*_1_, *s_j_*_2_, …, *s_jn_*) be two haplotypes of length *n*, where *s* is an SNP. If *s_il_*, *s_jl_* ≠ ‘–’ and *s_il_* ≠ *s_jl_*, then *h_i_* and *h_j_ mismatch* at locus *l*. If *s_il_*,*s_jl_* ≠ ‘*-*’ and *s_il_* = *s_jl_*, then *h_i_* and *h_j_ match* at locus *l*. Given a threshold *T_mis_*, [*p*, *q*] is a *maximum region of nearly identical matching* of *h_i_* and *h_j_* if *p* and *q* satisfy the following conditions:

(1) 1 ≤ *p* <*q* ≤ *n*;

(2) *h_i_* and *h_j_* match at loci *p* and *q*;

(3) there are no more than *T_mis_* continuous mismatches of *h_i_* and *h_j_* between *p* and *q*; and

(4) the number of matching loci of *h_i_* and *h_j_* in the region [*p*, *q*] is maximized.

To allow for genotyping errors, *T_mis_* should an integer greater than 0. When *T_mis_* is small, our experimental results (Table 2) show that our algorithm is robust with respect to the parameter. In our experiments, the default value of *T_mis_* is set to 2. Let the set of the maximum regions of nearly identical matching of *h_i_* and *h_j_* be *S_r_*(*h_i_*,*h_j_*), and the number of matching loci of *h_i_* and *h_j_* in a region [*p*, *q*] be *N_pq_*(*h_i_*,*h_j_*). The similarity measure of *h_i_* and *h_j_* is defined as(3)

**Table 2:** Experimental results when *T_mis_* varies

*T_mis_*	*Accuracy(%)*		

	1	2	3
HLA-A	95.89	96.50	96.50
HLA-B	93.57	94.31	94.26
HLA-C	94.82	96.65	96.34
HLA-DRB1	84.19	83.87	84.52
HLA-DQA1	98.00	98.29	98.29
HLA-DQB1	97.14	97.43	97.71

Accuracy of WSG-HI in the leave-one-out test using genotype data from the 200kb region centered around each HLA gene when the threshold *T_mis_* varies from 1 to 3 and *T_s_* = 0.65.

Intuitively, *Similarity*(*h_i_*, *h_j_*) reflects the likelihood that the two haplotypes *h_i_* and *h_j_* harbor a same HLA gene type. When two haplotypes *h_i_* and *h_j_* are identical, *Similarity*(*h_i_*, *h_j_*) reaches the maximum value 1, and the HLA gene types reside in these two haplotypes are equal with a high probability.

#### Weighted similarity graph

**Figure 7:**
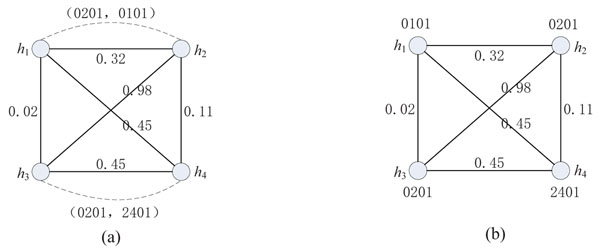
**An example of a weighted similarity graph and one of its feasible form** (a): An example of a weighted similarity graph. A solid line denotes a similarity edge and a dashed arc denotes a constraint edge. (b): A feasible form of (a)

Given a haplotype set *H* of a population inferred from the genotype matrix, a corresponding weighted similarity graph *G_H_* can be constructed as follows. For an individual with a pair of haplotypes {*h_i_*, *h_j_*} and a pair of HLA gene alleles {*α*,*β*}, there are two vertices *i* and *j* in *G_H_* and a *constraint edge c_ij_* between *i* and *j.* The edge *c_ij_* takes {*α*, *β*} as a *constraint*. If *α ≠ β*, *c_ij_* is a *heterozygous* constraint edge. If *α* = *β*, *c_ij_* is a *homozygous* constraint edge. Between any two vertices *p* and *q*, there is a *similarity edge e_pq_* taking a weight *w_pq_* = *Simlarity*(*h_p_*, *h_q_*). The set of vertices of *G_H_* is denoted as *V*(*G_H_*) and the set of HLA gene types in all constraints is denoted as *C*(*G_H_*). An example is given in Figure 7(a), where two individuals *I*_1_ and *I*_2_ are shown with their haplotypes {*h*_1_, *h*_2_} and {*h*_3_, *h*_4_}, as well as their HLA-A gene types {0201, 0101} and {0201, 2401}, respectively.

#### Optimal labeling of a weighted similarity graph

A labeling function *l* : *l*(*i*) = *α* defined on a similarity graph *G_H_* is a mapping from *V*(*G_H_*) to *C*(*G_H_*), where *i* ∈ *V*(*G_H_*) and *α* ∈ *C*(*G_H_*), which represents the assignment of an HLA gene type *α* to a vertex *i* (*i.e.*, its corresponding haplotype *h_i_*). For a constraint edge *c_ij_* with constraint {*α*, *β*}, if *l*(*i*) ∪ *l*(*j*) = {*α*, *β*}, the constraint edge is satisfied. When all constraints can be satisfied, the labeling function *l* is *feasible* and describes a Hap-HLA relation. A *feasible form* of *G_H_* is constructed by removing all the constraint edges (see Figure 7(b) for an example). A graph *G_H_* may have many different feasible forms that represent different Hap-HLA relations. To select an optimal one, a measure *Con* is introduced:(4)

where  denotes the set of vertices of . Given a weighted similarity graph *G_H_*, the optimal labeling problem is to find a feasible labeling *l* for *G_H_* such that  is maximized.

A naive brute-force search will take *O*(2*^N_h_^*) time for a graph *G_H_* with *N_h_* heterozygous constraint edges, which is impractical when *N_h_* is large. We develop a heuristic procedure *Heu-Label* (Additional file 3)to solve the problem. In Heu-Label, the vertices adjacent to homozygous constraint edges are labeled unambiguously in Step 2. In Step 3, we use a threshold *T_s_* to remove the similarity edges with small weights in *G_H_* and obtain a sparse graph *G.* We observed that when *T_s_* varies from 0.55 to 0.90, the performance of WSG-HI changed only slightly in our experiments (see Table 3). In the experimental tests, the default value of *T_s_* is set to 0.65. In the following steps, vertices are labeled in such a way that most vertices in a connected component of *G* are labeled by the same HLA gene type. The details are illustrated in Additional file 3. For briefness, the feasible form  of the weighted similarity graph *G_H_* obtained by the procedure Heu-Label is denoted by *G*(*H*).

**Table 3:** Experimental results when *T_s_* varies

*T_s_*	*Accuracy*(%)							

	0.55	0.60	0.65	0.70	0.75	0.80	0.85	0.90
HLA-A	95.89	95.89	96.50	95.89	95.89	95.89	95.89	95.89
HLA-B	95.00	94.64	94.31	94.64	94.64	94.64	94.64	94.64
HLA-C	96.34	96.34	96.65	95.73	96.04	95.43	95.12	93.29
HLA-DRB1	83.87	83.87	83.87	83.87	83.87	83.87	83.87	83.87
HLA-DQA1	98.29	98.29	98.29	97.71	98.00	97.71	97.71	96.57
HLA-DQB1	97.14	97.43	97.43	97.14	97.14	96.86	97.14	96.86

Accuracy of WSG-HI in the leave-one-out test using genotype data from the 200kb region centered at each HLA gene when the threshold *T_s_* varies from 0.55 to 0.90 and *T_mis_* = 2.

#### Optimal haplotype configurations and the Hap-HLA relation

In this subsection, we discuss the details of the incremental step of adding pedigrees with multiple haplotype solutions. Assume that an optimal haplotype configuration *H*′ and a Hap-HLA relation *l*′ of a subpopulation *P*′ have already been determined, which are encoded in the graph . When a new pedigree *P*″ is added to *P*′, the following procedure is used to search for an optimal haplotype assignment *H* and an optimal Hap-HLA relation *l* for *P*′ ∪ *P*″.

For the new pedigree *P*″, the extended DSS program uses a disjoint-set *D* to describe the solution space of haplotype assignments of *P*″. It can output a particular solution after assigning a particular value to a vector *S* = (*v*_1_, …,*v_f_*) of binary variables, where *f* is the number of free variables of the solution space. When the total number of different haplotype solutions of *P*″ is not larger than 2*^T_c_^* (*T_c_* = 10 in our experiments), a simple exhaustive enumeration procedure *Enum-Alg* illustrated in Additional file 4 is used to list all solutions one by one. For each haplotype solution *H*″ of *P*″, the procedure Enum-Alg adds new vertices and new edges to  and obtains an updated graph  for *P*′ ∪ *P*″. By applying the procedure Heu-Label to , we can obtain a new labeling *l* for the graph. Among all the solutions, Enum-Alg selects the solution that gives the maximum  as the assignment of pedigree *P*″ and also obtain an optimal Hap-HLA relation of *P*′ ∪ *P*″.

When the number of solutions is too large (*i.e.*, *f* > *T_c_*), we use a genetic algorithm denoted as *Genetic-Alg* to search for the optimal haplotype configuration and Hap-HLA relation. Genetic-Alg directly uses the solution variable *S* from the DSS algorithm to express an individual code, which uniquely represents a haplotype solution of *P*″, denoted as *H*(*S*). The fitness function of *S* is *Con*(*G*(*H*′ ∪ *H*(*S*))), where *H*′ is the haplotype configuration of *P*′ and *H*″ ∪ *H*(*S*) is the haplotype configuration of the population *P*′ ∪ *P*″. The hypothesis space is *H* = {(*v*_1_,*v*_2_,…,*v_f_*)|*v_i_* ∈ {0,1}, *i* = 1, 2,…, *f*}. Both tournament selection and roulette wheel selection [15] are adopted as genetic selection operators. To produce new individuals, single-point mutation and single-point crossover [15] are adopted. Please see Additional file 5 for details. In our experiments, the parameters of Genetic-Alg are set as follows: population size *p_s_* = 400, the maximum number of population generation *g_m_* = 50, crossover rate *r_c_* = 0.8, and mutation rate *r_m_* = 0.2.

#### HLA gene type inference

Let *R* be the set of the individuals whose HLA gene types are known and *U* the set of other individuals.

Let *H*(*I*) denote the haplotype pair of an individual *I*, *H*_1_(*I*) and *H*_2_(*I*) the haplotypes in *H*(*I*), and *V*_1_(*I*) and *V*_2_(*I*) the vertices in *G_H_* corresponding to *H*_1_(*I*) and *H*_2_(*I*) respectively. Let *w_m_*(*i*) denote the maximum weight of the similarity edges adjacent to vertex *i.* For a pair of HLA gene types {*g*_1_, *g*_2_}, let *w_m_*(*I*;*g*_1_, *g*_2_) denote

If after the procedure Heu-Label, the set *U* is still not empty, we use the following procedure to label vertices corresponding to an individual *I* ∈ *U*, based on the principle that similar haplotypes harbor similar HLA allele types. First, if *w_m_*(*V*_1_(*I*)) or *w_m_*(*V*_2_(*I*)) is smaller than the threshold *T_s_* (set to 0.65 in the experimental tests), the HLA gene types of *I* cannot be inferred. Otherwise, the HLA gene types of *I* can be inferred as follows. Let *L*(*V*_1_(*I*)) and *L*(*V*_2_(*I*)) be the set of labels of the vertices adjacent to *V*_1_(*I*) and *V*_2_(*I*) by the similarity edges with the maximum weights respectively, *i.e.*,

*L*(*V*_1_(*I*)) = {*l*(*p*) | *w*_*pV*_1_(*I*)_ = *w_m_*(*V*_1_(*I*)) ∧ *l*(*i*) ≠ ‘–’};

*L*(*V*_2_(*I*)) = {*l*(*p*) | *w*_*pV*_2_(*I*)_ = *w_m_*(*V*_2_(*I*)) ∧ *l*(*i*) ≠ ‘–’}.

If *L*(*V*_1_(*I*)) (or *L*(*V*_2_(*I*))) contains only one element, *V*_1_(*I*) (or *V*_2_(*I*)) is labeled by the element and the HLA gene type of *H*_1_(*I*) (or *H*_2_(*I*)) is determined. If there are more than one elements in *L*(*V*_1_ (*I*)) or *L*(*V*_2_(*I*)), HLA gene types *g*_1_ and *g*_2_ that satisfy the following condition are selected to label *V*_1_(*I*) and *V*_2_(*I*) respectively: *g*_1_ ∈ *L*(*V*_1_(*I*)), *g*_1_ ∈ *L*(*V*_2_(*I*)) and *w_m_*(*I;g*_1_,*g*_2_) is the maximum (see the procedure *HLA-type* in Additional file 6 for details).

#### Algorithm WSG-HI

The pseudocode of the algorithm WSG-HI is given as follows.

**INPUT:** genotype matrix *M_G_* and HLA matrix *M_H_* of a population *P* made up of pedigrees *p*_1_,…,*p_k_.*

**OUTPUT:** inferred HLA gene types for the individuals whose HLA gene types are unknown.

**STEP 1:** (find out an optimal Hap-HLA relation for *P*)

**Step 1.1**: **for***i* = 1,…, *k***do** apply the extended DSS to obtain a disjoint-set structure *D_i_* and free variables *v*_1_,…,*v_f_i__* that describe the solution space of the haplotype configuration of pedigree *p_i_;*

**Step 1.2**: sort the pedigrees in *P* in ascending order according to *f_i_*;

**Step 1.3:***H*′ = *P*′ = Ø; *i* = 1;

**Step 1.4: while***f_i_* = 0 and *i* ≤ *k***do** {*P*′ = *P*′ ∪*p_i_*; *H*′ = *H*′∪ the unique haplotype configuration *of p_i_; i* + +;}

**Step 1.5:** build a weight similarity graph *G_P_*_′_ for *P*′ using *M_G_* and *M_H_* ;

**Step 1.6:** apply procedure Heu-Label to *G_P_*_′_, and obtain *G*(*H*″) using *M_G_* and *M_H_;*

**Step 1.7: while***f_i_ < T_c_* and *i* ≤ *k***do**

{ *G*(*H*′) =Enum-Alg (*G*(*H*′),*p_i_*); *i* + +;}

**Step 1.8: while***i* ≤ *k***do**

{ *G*(*H*′) = Genetic-Alg(*G*(*H*′),*p_i_*); *i* + +;}

**Step 2:** (infer the HLA gene types for the individuals whose HLA gene types are unknown)

**Step 2.1:** scan *M_H_* to calculate the set *R* of the individuals in *P* whose HLA gene types are known and the set *U* of the other individuals;

**Step 2.2:** HLA-type(*G*(*H*′), *R*,*U*);

**Step 2.3:** the HLA gene types of an individual *I* ∈ *U* with the haplotype configuration (*h_p_*, *h_q_*) is ( *l* ( *p*), *l* (*q*))*;*

The running time of WSG-HI is mainly determined by the maximum size of the haplotype solution spaces of the pedigrees in the population *P.* When taking genotype data from the 200kb region as the input and running on a PC with Linux CentOS 5, 1GB memory and 2.4GHz CPU, WSG-HI takes 20 minutes to 1 hour to finish the test results for each HLA gene. Most of the time is used in the first step to compute an optimal Hap-HLA relation, and less than 0.01 seconds are used in Step 2. To check whether the performance is robust and stable, we tested WSG-HI five times using the leave-one-out strategy with the default parameter values. For each HLA gene, the tests exhibited very similar accuracy and running time (see Additional file 7 for the details).

## Competing interests

The authors declare that they have no competing interests.

## Authors contributions

MX designed the algorithm, performed the computational experiments. MX and JL drafted the manuscript. TJ supervised the project and polished the manuscript. All authors read and approved the manuscript.

## Supplementary Material

Additional file 1 The number of heterozygous SNPs in the regions around each HLA gene Click here for file

Additional file 2 The distribution of the number of different haplotype configurations obtained by applying the extended DSS algorithm to each pedigree of the CEU population. Click here for file

Additional file 3 The pseudocode of procedure Heu-Label. Click here for file

Additional file 4 The pseudocode of procedure Enum-Alg. Click here for file

Additional file 5 The pseudocode of procedure Genetic-Alg. Click here for file

Additional file 6 The pseudocode of procedure HLA-type. Click here for file

Additional file 7 Accuracy and running time of WSG-HI tested repeatedly five times. Click here for file
