# Supplementary material for: Accurate HLA type inference using a weighted similarity graph
Source: BMC Bioinformatics. 2010 Dec 14;11(Suppl 11):S10. doi: 10.1186/1471-2105-11-S11-S10 (PMC3024871; doi:10.1186/1471-2105-11-S11-S10)
Supplement: Additional file 1 — The number of heterozygous SNPs in the regions around each HLA gene [file 1471-2105-11-S11-S10-S1.pdf]

The number of heterozygous SNPs in the regions around each HLA gene

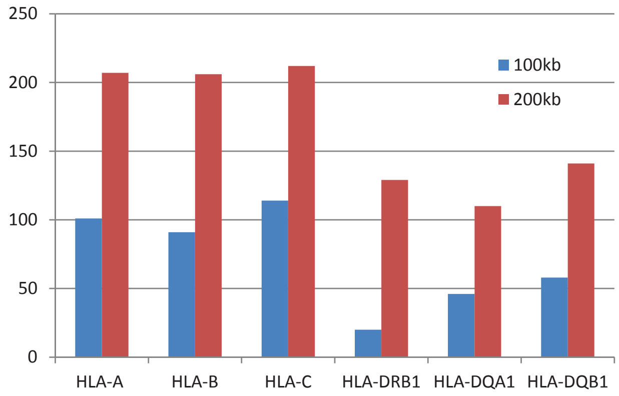

The number of SNPs in the regions around each HLA gene. The vertical axis shows the numbers of heterozygous SNPs in the 100kb regions (blue bars) and the 200kb (red bars) centered at each HLA gene.
