# Supplementary material for: Accurate HLA type inference using a weighted similarity graph
Source: BMC Bioinformatics. 2010 Dec 14;11(Suppl 11):S10. doi: 10.1186/1471-2105-11-S11-S10 (PMC3024871; doi:10.1186/1471-2105-11-S11-S10)
Supplement: Additional file 2 — The distribution of the number of different haplotype configurations obtained by applying the extended DSS algorithm to each pedigree of the CEU population. [file 1471-2105-11-S11-S10-S2.pdf]

The distribution of the number of different haplotype configurations

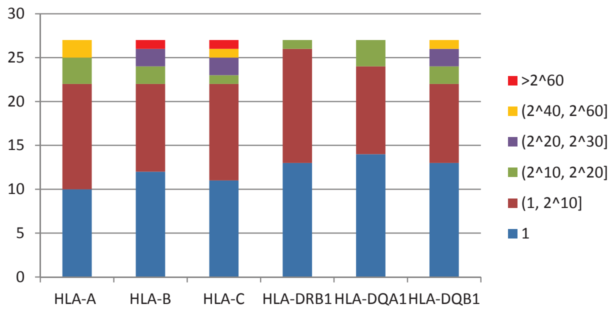

(a) 100kb regions

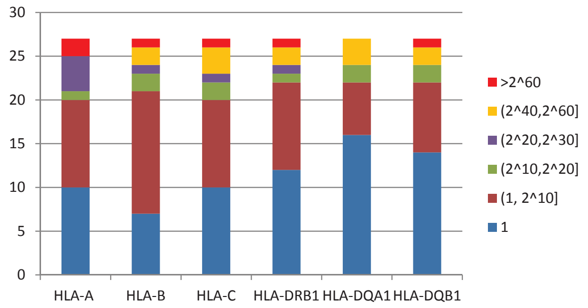

(b) 200kb regions

The distribution of the number of different haplotype configurations obtained by applying the extended DSS algorithm to each pedigree of the CEU population. The genotype data are extracted from the file http://www.inflammgen.org/inflammgen/files/data/CEU+.pedfile.txt, which contains 27 pedigrees. Given genotype data from the 100kb regions (a) or in the 200kb regions (b) centered at six HLA genes, the number of pedigrees whose haplotype solution numbers are in a specific range is represented by the height of a colored bar. For example, in the column labeled as HLA-A, the height of the blue bar shows that given genotype data from the 100kb region around the HLA-A gene, there are 10 pedigrees that have a unique haplotype solution, and the height of the yellow bar shows that there are two pedigrees whose numbers of haplotype solutions are between 2^{40} and 2^{60}.
