# Supplementary material for: Accurate HLA type inference using a weighted similarity graph
Source: BMC Bioinformatics. 2010 Dec 14;11(Suppl 11):S10. doi: 10.1186/1471-2105-11-S11-S10 (PMC3024871; doi:10.1186/1471-2105-11-S11-S10)
Supplement: Additional file 3 — The pseudocode of procedure Heu-Label. [file 1471-2105-11-S11-S10-S3.pdf]

The pseudocode of procedure Heu-Label

Input: A weighted similarity graph G_H.
Output: A labeling l of G_H.
Step 1: **for** each vertex i of G_H **do** { $l(i) =$ '$-$'; }
Step 2: **for** each homozygous constraint edge c_{ij} of G_H **do**
$\alpha =$ an HLA gene type in the constraint of c_{ij}; $l(i) = l(j) = \alpha$; delete c_{ij} from G_H;
Step 3: build a graph G by deleting all constraint edges and the similarity edges whose weights are small than T_s from G_H;
Step 4: find all connected components of G by depth first search;
Step 5: **for** each connected component *comp* (from the largest to the smallest) **do**
Step 5.1: **for** each HLA gene type α in $C(G_H)$ **do** { $N(\alpha) = 0$;}
Step 5.2: **for** each vertex i in *comp* **do**
if $l(i) \neq$ '$-$' **then** $N(l(i)) + +$;
else { $(\alpha, \beta) =$ the constraint of the constraint edge adjacent to i in G_H; $N(\alpha){+}{+}$; $N(\beta){+}{+}$;}
Step 5.3: $\gamma = \underset{\alpha}{\operatorname{argmax}}(N(\alpha))$;
Step 5.4: **for** each vertex i in *comp* **do**
$(\alpha, \beta) =$ the constraint of the constraint edge c_{ij} adjacent to i in G_H;
if $l(i) =$ '$-$' **then**
if $\alpha = \gamma$ **then** $l(i) = \alpha$; $l(j) = \beta$; delete the constraint edge c_{ij} from G_H;
delete vertices i and j from G;
if $\beta = \gamma$ **then** $l(i) = \beta$; $l(j) = \alpha$; delete the constraint edge c_{ij} from G_H;
delete vertices i and j from G;
Step 6: repeat Steps 4 and 5 until there are no more vertices can be labeled.
