# Supplementary material for: Accurate HLA type inference using a weighted similarity graph
Source: BMC Bioinformatics. 2010 Dec 14;11(Suppl 11):S10. doi: 10.1186/1471-2105-11-S11-S10 (PMC3024871; doi:10.1186/1471-2105-11-S11-S10)
Supplement: Additional file 4 — The pseudocode of procedure Enum-Alg. [file 1471-2105-11-S11-S10-S4.pdf]

The pseudocode of procedure Enum-Alg

Enum-Alg($G^{l'}_{H'}$, P'')
{ **for** each haplotype configuration H'' of P'' **do**
{ construct a graph $G^{l'}_{H' \cup H''}$ by adding two vertices for each individual of P'', the corresponding similarity edges and constraint edges in $G^{l'}_{H'}$;
apply **Heu-Label** to $G^{l'}_{H' \cup H''}$ to obtain a labeling l for $G^{l'}_{H' \cup H''}$;
}
return the $G^{l}_{H' \cup H''}$ with the maximum $Con(G^{l}_{H' \cup H''})$;
}
