# Supplementary material for: Accurate HLA type inference using a weighted similarity graph
Source: BMC Bioinformatics. 2010 Dec 14;11(Suppl 11):S10. doi: 10.1186/1471-2105-11-S11-S10 (PMC3024871; doi:10.1186/1471-2105-11-S11-S10)
Supplement: Additional file 5 — The pseudocode of procedure Genetic-Alg. [file 1471-2105-11-S11-S10-S5.pdf]

The pseudocode of procedure Genetic-Alg

Genetic-Alg($G^{l'}_{H'}$, P'')
{ $//p_s$: population size, r_c: crossover rate, r_m: mutation rate.
$//g_m$: the maximum number of population generation.
$//f$: the number of free variables to describe the haplotype configuration space of P''.
randomly generate p_s individuals (*i.e.*, f dimensions vector of {0,1}) to form a population $\mathcal{P}_0$;
for $i = 1$ to g_m **do**
{ **for** each individual $\mathcal{S} \in \mathcal{P}_0$ **do**
{ compute the fitness of $\mathcal{S}$, *i.e.*, $Con(G(H' \cup H(\mathcal{S})))$;
if the fitness of $\mathcal{S}$ is maximum so far **then** $\mathcal{S}_m = \mathcal{S}$;
}
if the fitness of $\mathcal{S}_m$ remains unchanged or $i = g_m$ **then return** $G(H' \cup H(\mathcal{S}_m))$;
// produce a new generation of population
$\mathcal{P}_1 = \varnothing$;
select $(1 - r_c) \times p_s$ individuals from P_0 into P_1 using the tournament selection operator;
select $r_c \times p_s/2$ pairs of individuals from P_0 using the roulette wheel selection operator into $\mathcal{M}$;
for each pair $(i, j) \in \mathcal{M}$ **do**
{ randomly apply single-point crossover operator on (i, j) and put the two offspring into P_1; }
select $r_m \times p_s$ members from P_1 randomly and invert the value at a random position for each selected member;
$\mathcal{P}_0 = \mathcal{P}_1$;
}
}
