# Supplementary material for: Accurate HLA type inference using a weighted similarity graph
Source: BMC Bioinformatics. 2010 Dec 14;11(Suppl 11):S10. doi: 10.1186/1471-2105-11-S11-S10 (PMC3024871; doi:10.1186/1471-2105-11-S11-S10)
Supplement: Additional file 6 — The pseudocode of procedure HLA-type. [file 1471-2105-11-S11-S10-S6.pdf]

The pseudocode of procedure HLA-type

HLA-type(G_H, R, U)
{ **for** each $I \in U$ **do**
{ $i = V_1(I)$; $j = V_2(I)$;
$w_m(i) = \max_k(w_{ik})$;
$w_m(j) = \max_k(w_{jk})$;
$L(i) = L(j) = \varnothing$;
if $w_m(i) < T_s$ or $w_m(j) < T_s$ **then**
{ $l(i) =$ '$-$'; $l(j) =$ '$-$'; continue; }
for each vertex $k \in R$ **do**
{ **if** $w_{ik} = w_m(i)$ **then** $L(i) = L(i) \cup l(k)$;
if $w_{jk} = w_m(j)$ **then** $L(j) = L(j) \cup l(k)$;
}
if $|L(i)| = 1$ **then** $l(i) =$ the element in $L(i)$;
if $|L(j)| = 1$ **then** $l(j) =$ the element in $L(j)$;
if $|L(i)| > 1$ or $|L(j)| > 1$ **then**
{ traverse all constraint edges of G_H to compute $w_m(I; g_p, g_q)$ for $g_p \in L(i)$ and $g_q \in L(j)$;
$(g_1, g_2) = \operatorname*{argmax}_{g_p \in L(i), g_q \in L(j)} w_m(I; g_p, g_q)$;
$l(i) = g_1; l(j) = g_2$;
}
}
}
