# Supplementary material for: Accurate HLA type inference using a weighted similarity graph
Source: BMC Bioinformatics. 2010 Dec 14;11(Suppl 11):S10. doi: 10.1186/1471-2105-11-S11-S10 (PMC3024871; doi:10.1186/1471-2105-11-S11-S10)
Supplement: Additional file 7 — Accuracy and running time of WSG-HI tested repeatedly five times. [file 1471-2105-11-S11-S10-S7.pdf]

Experimental results of five repeated tests

Accuracy and running time of WSG-HI tested repeatedly five times using the leave-one-out strategy and genotype data from the 200kb region centered at each HLA gene with T_s = 0.55 and T_{mis} = 2.

Gene	Accuracy (%)					Running time (min)				
	1	2	3	4	5	1	2	3	4	5
**HLAA**	96.50	96.20	96.82	96.50	96.20	55.5	56.4	56.3	56.2	56.1
**HLAB**	94.30	94.66	95.00	95.00	95.00	47.3	47.6	47.7	47.6	47.6
**HLAC**	96.65	96.65	96.65	96.34	96.34	58.9	59.7	59.8	59.9	59.0
**HLADRB1**	83.87	84.19	84.19	83.87	83.87	41.6	41.6	41.5	41.5	41.5
**HLADQA1**	98.29	98.57	98.29	98.57	98.57	29.8	29.7	29.7	29.8	29.7
**HLADQB1**	97.43	97.71	97.71	97.43	98.00	37.3	38.1	38.1	38.1	38.1
